# Supplementary material for: Predicting the Performance of Concurrent Systematic Random Biopsies during Image Fusion Targeted Sampling of Multi-Parametric MRI Detected Prostate Cancer. A Prospective Study (PRESET Study)
Source: Cancers (Basel). 2021 Dec 21;14(1):1. doi: 10.3390/cancers14010001 (PMC8750557; doi:10.3390/cancers14010001)

Table S1: MRI acquisition parameters

	T1WI	High resolution T2WI			DWI		DCE
	Axial	Sagittal	Axial	Coronal	DWI	DWI high b-value	Dyn Gd-MRI
**Sequence**	2DTSE	2DTSE	2DTSE	2DTSE	2DEPI	2DEPI	3D VIBE
**TR (ms)**	650	6000	4000	5000	3300	3300	4.76
**TE (ms)**	11	102	100	100	95	95	2.45
**Flip angle (°)**	150	140	150	150	–	–	10
**Slice thickness (mm)**	3	3	3	3	3	3	3
**Slice gap (mm)**	0.6	0.6	0.6	0.6	0	0	0.6
**Resolution (pixels)**	320	320	320	320	192	192	192
**FOV (mm)**	200	200	200	200	280	280	280
**b-values (s/mm²)**	–	–	–	–	50,100,500,1000	2000	–
**Temporal resolution (s)**	–	–	–	–	–	–	4

Figure S1: Receiver operating characteristics (ROC) curve and area under the curve (AUC) for model discriminative ability

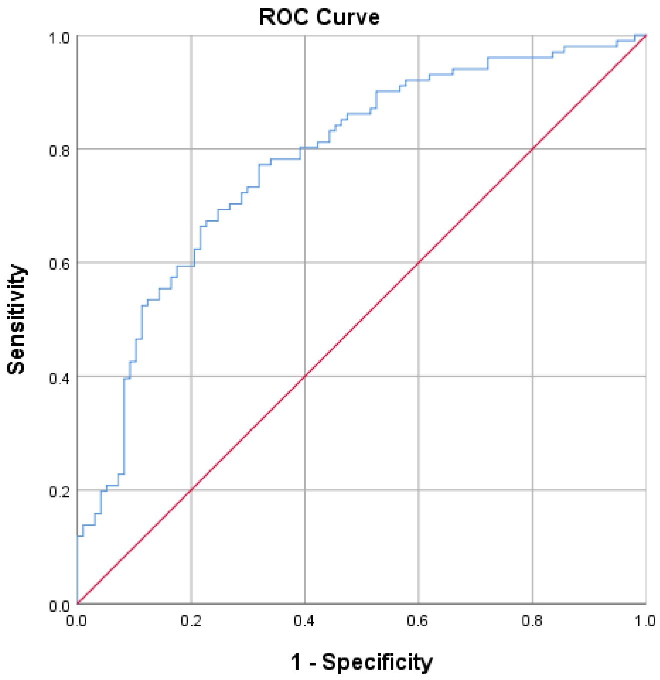

Supplement: Supplementary file 1 [file cancers-14-00001-s001.zip › cancers-1503666-supplementary.pdf]
